# Comparative Efficacy of Combined Carbon Dioxide Fractional Laser and Pulse Dye Laser versus Monotherapy for Hypertrophic Scars: A Network Meta-Analysis of Randomized Controlled Trials

**DOI:** 10.1007/s00266-026-05829-9

**Published:** 2026-04-15

**Authors:** Derong He, Jingjing Zhang, Baiye Chen, Chaofan Lin, Weiwen Zhu, Qingcheng Liu, Xunyu Xu

**Affiliations:** 1 Department of Plastic Surgery, Fujian Children’s Hospital (Fujian Branch of Shanghai Children’s Medical Center), College of Clinical Medicine for Obstetrics and Gynecology and Pediatrics, Fujian Medical University, Fuzhou, Fujian China; 2 Department of Thoracic Surgery, College of Clinical Medicine of Fujian Provincial Hospital, Fujian Medical University, No. 134, Dongjie, Gulou District, Fuzhou, 350001 Fujian China

**Keywords:** Proliferative scarring, Carbon dioxide fractional laser, Pulsed dye laser, Combination therapy, Network Meta-analysis

## Abstract

**Background:**

The efficacy of segmental carbon dioxide laser (FCO_2_L) combined with pulsed dye laser (PDL) treatment versus single laser treatment for hypertrophic scars has not been fully evaluated. This study conducted a network meta-analysis to compare the efficacy of the combined treatment with that of the single laser treatment.

**Methods:**

PubMed, Embase, Cochrane Library, and Web of Science databases were systematically searched up to 28 April 2025. Two independent reviewers conducted study selection, data extraction, and risk-of-bias assessment. The quality of the included studies was evaluated using the Risk of Bias (RoB) 2.0 tool. A Bayesian network meta-analysis model was employed to calculate the standardized mean difference (SMD) and corresponding 95% confidence intervals (CIs) for VSS scores. Treatment rankings were determined using the surface under the cumulative ranking curve (SUCRA).

**Results:**

A total of 14 randomized controlled trials were included, involving 326 patients. The network meta-analysis showed that the therapeutic effect of FCO_2_L combined with PDL was significantly better than that of the single therapy. Specifically: Compared with single PDL: SMD =  − 1.29 (95% CI:  − 1.94,  − 0.65). The SUCRA probability ranking showed that the combined ranking of FCO_2_L combined with PDL was the highest (probability > 80%), followed by single FCO_2_L (70%-75%).

**Conclusion:**

The combined use of FCO_2_L and PDL has significant advantages in improving hypertrophic scars. Although using FCO_2_L alone is effective, its therapeutic effect is not as good as the combined strategy.

**Level of Evidence I:**

This journal requires that authors assign a level of evidence to each article. For a full description of these Evidence-Based Medicine ratings, please refer to the Table of Contents or the online Instructions to Authorswww.springer.com/00266.

**Supplementary Information:**

The online version contains supplementary material available at 10.1007/s00266-026-05829-9.

## Introduction

Hyperproliferative scar (HS) is a common complication of poor wound healing after skin damage, usually secondary to trauma, burns, skin infections, and surgery. It is caused by excessive stress response after skin damage, and its pathological features are mainly manifested as excessive value-added, deposition, and disorder of collagen, fibroblasts, and extracellular matrix [1, 2]. Currently, the commonly used treatment methods for hypertrophic scars in clinical practice include topical or intramuscular application of glucocorticoids, intense pulsed light therapy, carbon dioxide fractional laser therapy or pulsed dye laser therapy, pressure therapy, radiotherapy, surgical excision, silicone products, and cryotherapy [3, 4]. Since scarring is not as effective as it should be, with HS chronically prolonged for decades and prone to recurrence, combination therapy strategies of various therapies are favored by clinical practitioners. Currently, combined treatment options with lasers have made some progress in the clinic [5], but fewer studies have evaluated the effectiveness of the combination of carbon dioxide fractional laser and pulsed dye laser versus single laser treatment.

The CO_2_ fractional laser, with a wavelength of 10600 nm, is a clinically used laser instrument based on selective photothermal action [4]. The CO_2_ fractional laser is used to treat hypertrophic scarring by scanning the laser light in a sequential fractional pattern and using the uniform column of microporous channels formed by the laser light to stimulate the regeneration and rearrangement of subcutaneous collagen and improve hypertrophic scarring [6, 7]. Pulsed dye laser has a special wavelength of 585-595nm, which is also based on the principle of selective photothermal action, and the target tissue is the hemoglobin of the blood vessel wall. It can penetrate the epidermis and dermis, reaching the local area where the diseased blood vessels are located, thereby achieving the inhibition and destruction of abnormal dilated vascular tissues, and ultimately achieving the goal of closing the local blood vessels [8, 9]. In addition, pulsed dye laser can also cut off the nutrient supply in the lesions of vasoproliferative diseases, further preventing the reperfusion of abnormal blood vessels and avoiding the progress of the disease. Existing studies have shown that both segmental carbon dioxide (FCO_2_L) laser and pulsed dye laser (PDL) are effective in reducing the VSS (Vancouver Scar Scale) score and VAS (Visual Analogue Scale) pain score [10, 11], but there is still a lack of head-to-head meta-analyses of the efficacy of fractional carbon dioxide laser, pulsed dye laser, and the combination of these two methods in the treatment of proliferative scarring. However, in the field of treating hypertrophic scars, there is currently a lack of head-to-head comparative meta-analysis studies on carbon dioxide fractional laser, pulsed dye laser, and their combined therapies. Therefore, it is difficult to directly evaluate the specific efficacy of these three treatment options in the treatment of hypertrophic scars.

This study aims to evaluate the efficacy of these two laser therapies and their combined therapies in the treatment of hypertrophic scars. To this end, we conducted a network meta-analysis to assess the therapeutic effects of segmental carbon dioxide laser, pulsed dye laser, and carbon dioxide laser combined with pulsed dye laser in the treatment of hypertrophic scars, thereby providing a reference for the selection of clinical treatment plans and suggesting future research directions.

## Methods

### Search Strategy

To conduct this study, a comprehensive search was carried out in four major databases: PubMed, Embase, Cochrane Library, and Web of Science. The search period covered from the inception date of each database to April 28, 2025. During the literature search process, researchers used the following keyword combinations for the search: ("hypertrophic scar") AND ("fractional carbon dioxide laser" OR "pulsed dye laser" OR "laser therapy") AND ("randomized controlled trial" OR "clinical controlled trial" OR "placebo" OR "random allocation" OR "randomization"). Additionally, to further refine the research content, we also carefully reviewed the reference lists of the included literature to supplement any potentially missed relevant studies. The detailed database search strategy is presented in Supplementary Table 1.

### Study Selection Criteria

Inclusion criteria were as follows: (i) Subjects: Patients with hypertrophic scars, regardless of age, location of the lesion, race, nationality, or gender, if they have no surgical contraindications and can fully participate in the follow-up and the study, are eligible to participate. (ii) Intervention: Treatment of hypertrophic scarring with laser only. (iii) Outcome indicators: The primary outcome measure pre-specified for this network meta-analysis was the change in total Vancouver Scar Scale (VSS) score from baseline to post-treatment. The VSS is a comprehensive scale evaluating scar color, vascularity, thickness, and flexibility, with higher scores indicating more severe scarring. Combining the continuous variable of VSS score change allows for a more nuanced comparison of treatment efficacy. Some studies also reported Patient and Observer Scar Assessment Scale (POSAS) or Visual Analog Scale (VAS) scores. However, due to data availability and consistency, this analysis primarily relies on VSS as the comparative benchmark. (iv) Type of study: All relevant studies included are randomized controlled trials, and the study data need to contain valid primary data such as the effective rate of laser treatment.

Exclusion criteria were as follows: (i) non-randomized controlled studies. (ii) Studies for replication of literature, systematic evaluations, animal experiments, reviews, and case reports. (iii) Studies of the concurrent use of other treatments for proliferative scarring, such as surgery, injections, local radiotherapy, or compression therapy. (iv) Evaluation indicators for clinical studies do not include laser treatment efficacy rates or valid raw data that cannot be calculated.

### Data Extraction

Two researchers independently reviewed titles and abstracts to identify studies relevant to the article topic. Eligible literature was downloaded in full and assessed by two independent evaluators. The data for the study were extracted by two independent evaluators. The extracted data included: (1) authors; (2) publication year; (3) country; (4) participants (age, gender, sample size); (5) characteristics of the intervention measures (intervention type); and (6) results (laser treatment effect). In case of any discrepancies during this process, consensus among the evaluators will be reached to resolve them. If necessary, a third evaluator can also be consulted.

### Risk-of-Bias Assessment

Reviewers independently duplicated the work and assessed risk of bias using a revised version of the Cochrane Risk Assessment Tool. Risk of bias was assessed using a revised version of the Cochrane Risk of Bias Assessment Tool for Randomized Trials (RoB 2.0) [12, 13]. The evaluation factors include bias during the randomization process, deviations from the expected intervention measures, the absence of result data, result measurement, and the selection of the intervention subjects, etc. The assessment results of the risk of bias are low, possibly low, possibly high, or high. When necessary, differences can be resolved through discussion and third-party adjudication.

### Statistical Analysis

A network meta-analysis (NMA) was performed to synthesize direct and indirect evidence across all included interventions. First, a network geometry plot was constructed to visualize the evidence base, where each node represented an intervention, and the edges between nodes indicated the availability of direct head-to-head comparisons. The width of edges was proportional to the number of studies for each comparison. We employed a frequentist random-effects NMA model under the assumption of consistency to estimate the relative effects between all interventions. This model accounts for heterogeneity across studies beyond within-study sampling error. Heterogeneity within the entire network was assessed using the I^2^ statistic, interpreted as follows: 0%–30%, insignificant; 30%–50%, moderate; 50%–75%, significant; and 75%–100%, highly significant. The consistency assumption (i.e., agreement between direct and indirect evidence) was evaluated globally and locally. Local inconsistency for each paired comparison was assessed using the node-splitting method, where a *P* value > 0.05 suggested no significant discrepancy between direct and indirect estimates. The results are presented as standardized mean differences (SMDs) with 95% confidence intervals (CIs) for the primary outcome (VSS score reduction). All pairwise comparisons are summarized in a league table, and the relative effects against a common reference (e.g., no laser treatment) are displayed in a forest plot. Treatment rankings were estimated using the surface under the cumulative ranking curve (SUCRA) and mean ranks. Higher SUCRA values (range 0%-100%) indicate a greater probability of being the most effective intervention. Potential publication bias or small-study effects were examined visually using a comparison-adjusted funnel plot and statistically assessed via Egger’s regression test. A *P* value > 0.05 was considered indicative of no significant asymmetry. All analyses were conducted using R software (version 4.2.3) with the netmeta package. Statistical significance was set at a two-sided *P* < 0.05.

## Results

### Study Selection and Study Characteristics

**Fig. 1:**
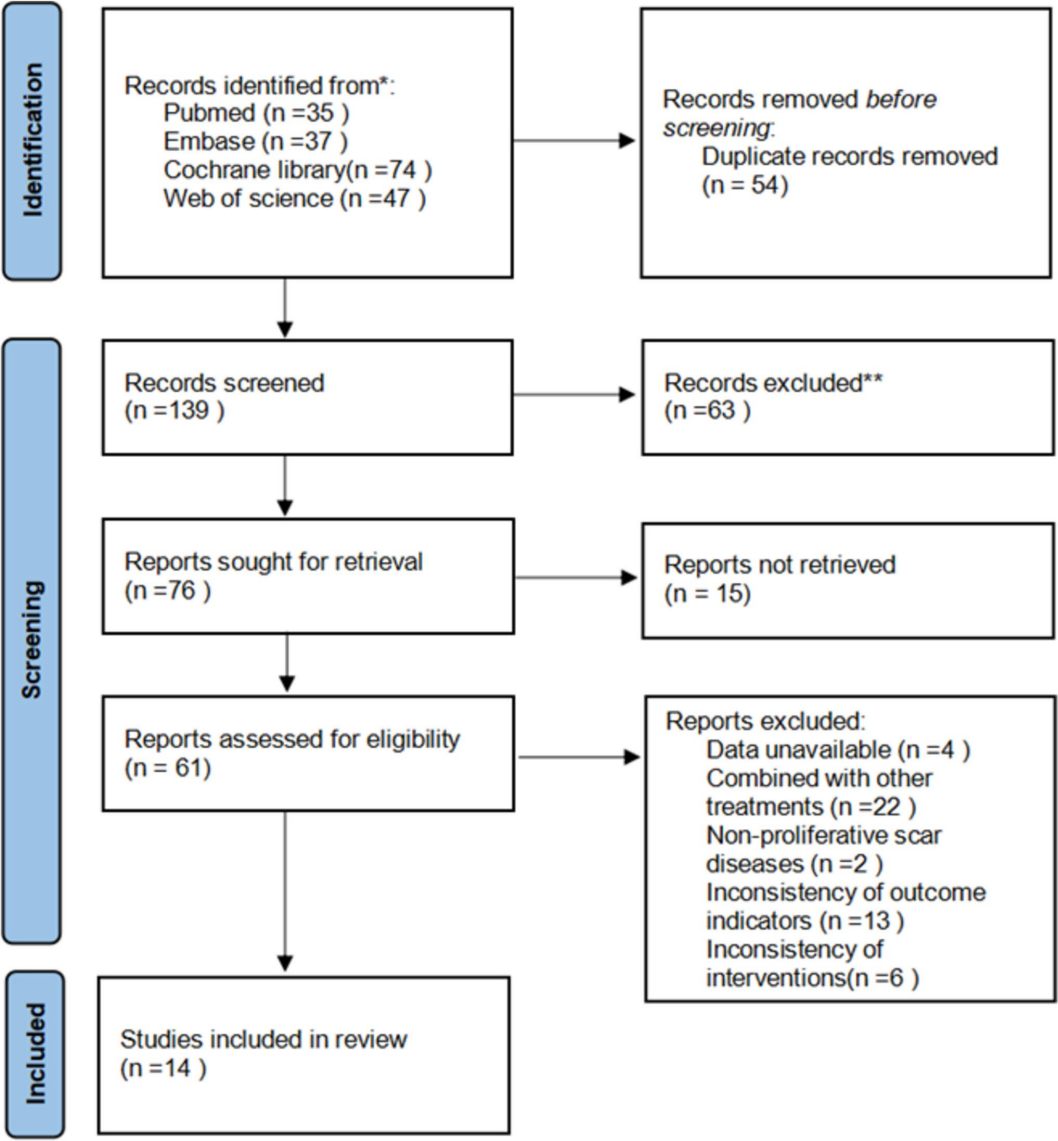
Literature screening flowchart

A total of 193 studies were identified through database searches. After removing duplicates, 139 papers were screened. After review of titles and abstracts, 63 entries were excluded, leaving 76 papers for full-text analysis. Ultimately, 14 studies and 326 participants were included in the network meta-analysis (Fig. 1).

Finally, 14 studies were included [10, 14–26], including nine experimental reports on laser therapy, namely FCO_2_L combined with PDL, FCO_2_L, PDL, microneedling, intense pulsed light, potassium titanyl phosphate laser, long pulse Nd, FCO_2_L combined with long pulse Nd, and FCO_2_L combined with PDL. The study designs were all randomized controlled studies. Most of the studies were conducted in Europe and Africa (71.4%). The majority of patients were between 16 and 66.3 years of age, with similar proportions of male and female patients (Table 1).

**Table 1:** Characteristics of included randomized controlled trials

Study	Year	Country	Age, yr, mean	Sex, male,%	Intervention	Comparator	No. of patients		
							Total	Trail	Control
Laura E	2022	American	29	76	CO_2_+PDL	CO_2_ or PDL	25	25	25
Samia Esmat	2021	Egypt	NR	NR	CO_2_	Microneedling	30	15	15
Christopher J	2023	Australia	42	80	CO_2_	Control (no laser treatment)	15	15	15
Terrence C	2016	American	50	5	PDL	KTP laser treatment	20	10	10
Ouyang	2017	China	28	62.5	CO_2_+PDL	PDL	56	28	28
Alexander A	2019	American	36	NR	CO_2_	IPL /Control (no laser treatment)	19	19	19
Shereen O	2020	Egypt	30	26.7	CO_2_	long‐pulsed Nd/CO_2_+long‐pulsed Nd	30	30	30
Zaynab Sayed	2025	Egypt	16	43.3	CO_2_	Control (no laser treatment)	30	30	30
Shereen O	2022	Egypt	24.8	10	CO_2_	Microneedling	20	20	20
K.P. Allison	2002	UK	NR	NR	PDL	Control (no laser treatment)	38	38	38
Yang	2012	China	26.4	46	PDL	Control (no laser treatment)	26	26	26
Padcha	2019	Thailand	66.3	13	PDL	Control (no laser treatment)	40	40	40
Maedeh Karimi	2024	Tehran	23.4	20	PDL	CO_2_/PDL+CO_2_	20	20	20
O. A. Azzam	2015	Egypt	24.5	61	CO_2_	Control (no laser treatment)	12	12	12

*CO*_*2*_ Carbon dioxide (CO_2_) fractional ablative laser, *PDL* Pulse dye laser, *IPL* Intense pulsed light, *KTP* The potassium titanyl phosphate laser, *NR* Not reported

### Risk-of-Bias Assessment

**Fig. 2:**
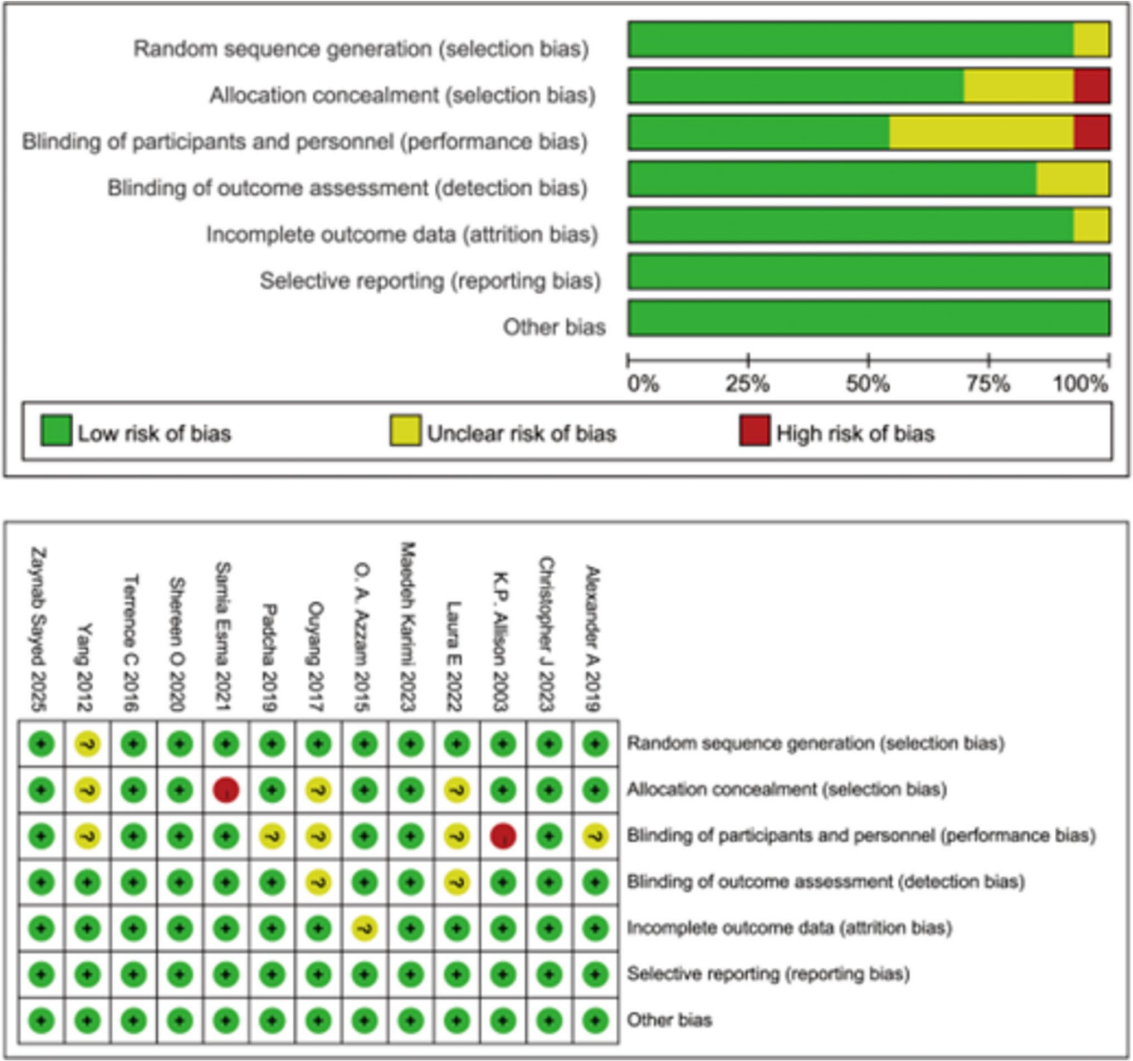
Risk assessment Chart

The results of the risk-of-bias assessment for the included studies are shown in Fig. 2. Overall, the risk of bias was low in 11 studies, while the risk of bias was unclear in 3 studies.

### Network Meta-Analysis

**Fig. 3:**
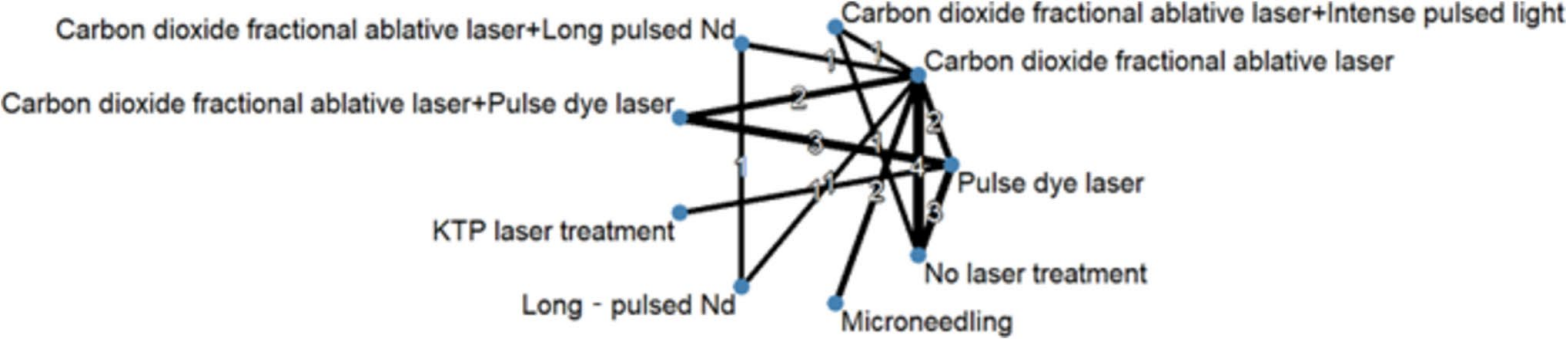
Full network evidence plot for VSS scores. Nodes represent interventions, with size proportional to the number of patients assigned to that intervention. Connections represent direct comparisons, with thickness proportional to the number of studies included in the comparison

Figure 3 presents a network diagram comparing VSS scores for different laser therapies treating hypertrophic scars. This well-connected evidence network permits indirect comparative analysis.

**Fig. 4:**
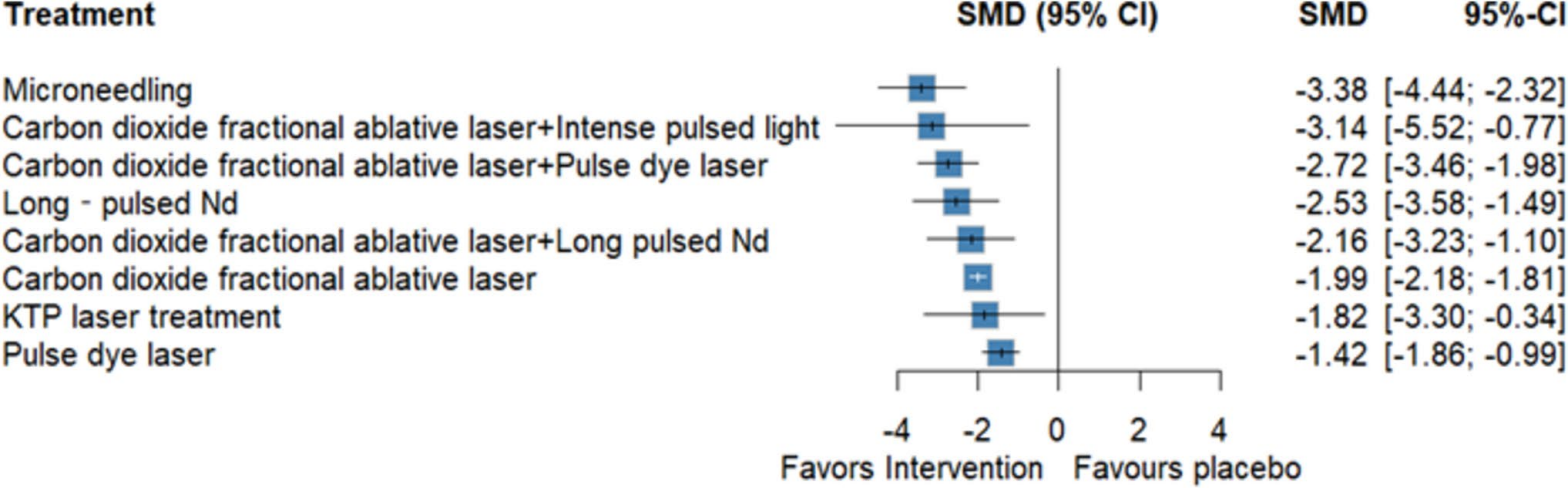
Forest plot for VSS scores

**Fig. 5:**
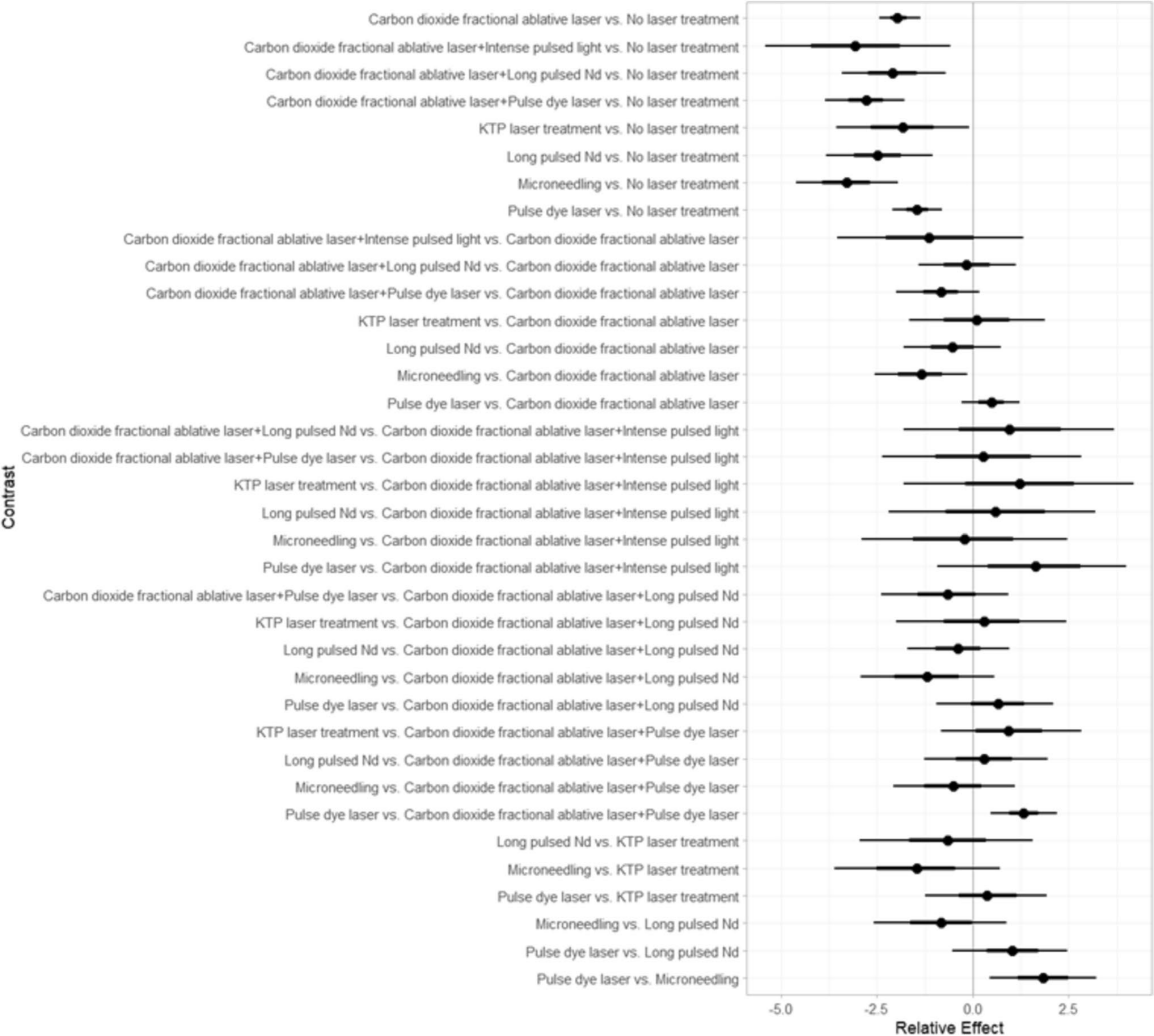
Node segmentation chart

This study conducted a network meta-analysis to compare the efficacy of various laser therapy regimens for hypertrophic scars. Using the VSS score reduction as the primary outcome, the SMD and its 95% CI indicate that the combination of CO_2_ fractional laser and pulsed dye laser outperformed either monotherapy or no laser treatment in reducing VSS scores for hypertrophic scars. Specifically, the SMD for the combination therapy versus monotherapy with pulsed dye laser was  − 1.29 (95% CI:  − 1.94,  − 0.65), indicating significantly greater improvement in scar severity (VSS score) with the combination approach (Figs. 4 and 5). Furthermore, the direct estimated SMD for combined therapy versus no laser treatment ( − 2.72, 95% CI:  − 3.46,  − 1.98) was consistent with the network estimation results (SMD:  − 2.72, 95% CI:  − 3.46,  − 1.98), further supporting its therapeutic advantage in reducing VSS scores (Table 2).

**Table 2:**
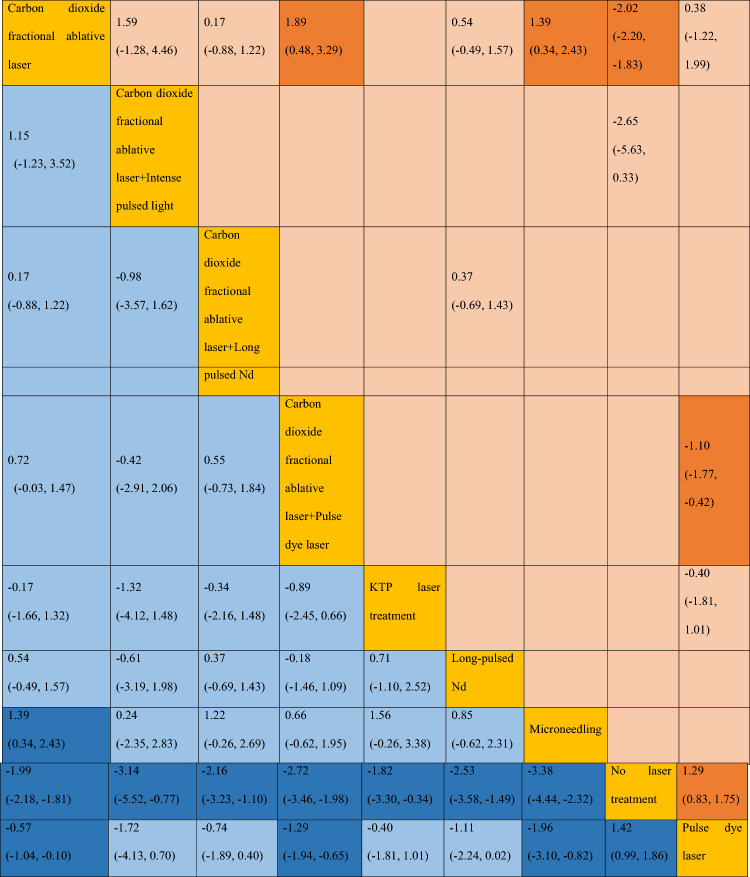
League table

Results from the network meta-analysis (mixed [network] and indirect comparisons) are presented in the lower left triangle, and results from pairwise meta-analyses (direct comparisons) are presented in the upper right triangle. Deeper background color means statistically significant. The data are the standardized mean difference (95% CrI), and data above 0 favor the line-defining treatment.

In monotherapy, the network-estimated SMD for CO_2_ fractional laser monotherapy versus no laser treatment was  − 1.99 (95% CI:  − 2.18,  − 1.81), while the SMD for pulsed dye laser monotherapy versus no laser treatment was  − 1.42 (95% CI:  − 1.86,  − 0.99). Both demonstrated efficacy in reducing VSS scores, though their effect sizes were smaller than those observed with combination therapy. Among other combination regimens, CO_2_ fractional laser combined with intense pulsed light (FCO_2_L+IPL) showed an SMD of  − 3.14 (95% CI:  − 5.52,  − 0.77) compared to no laser treatment; however, this result should be interpreted with caution due to the wide confidence interval (Fig. 5).

### Treatment Ranking and Probability Analysis

**Fig. 6:**
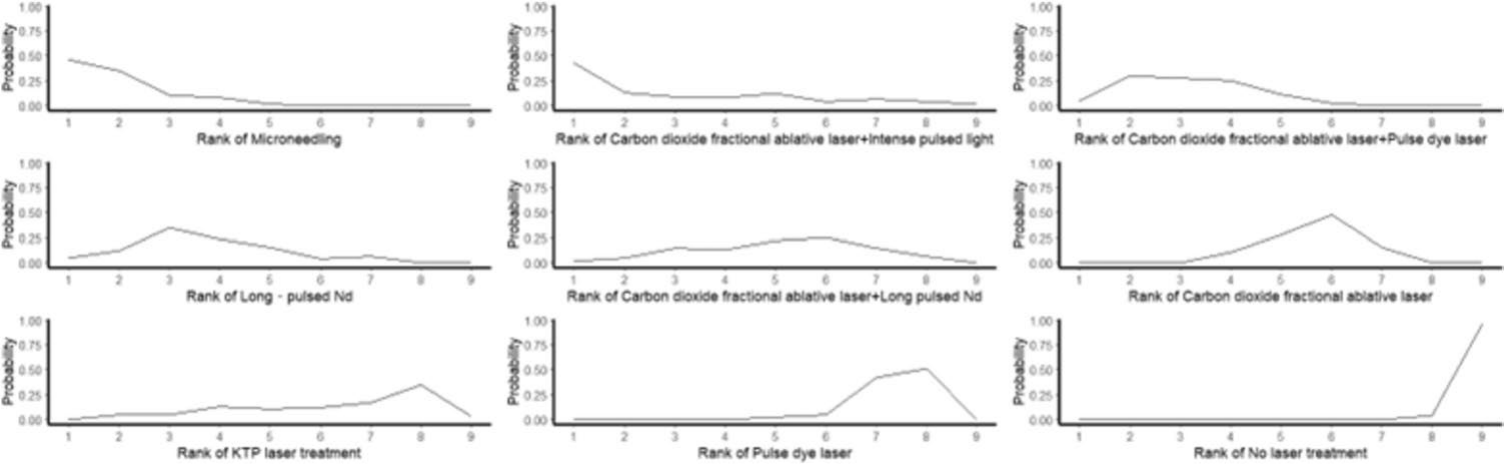
SUCRA sorting chart

Probability-based ranking using the SUCRA revealed that fractional CO_2_ laser combined with pulsed dye laser (FCO_2_L+PDL) achieved the highest overall ranking (SUCRA value >80%), indicating that it is most likely to be the optimal intervention for reducing VSS scores (Fig. 6). CO_2_ fractional laser monotherapy (SUCRA value: 70%–75%) and CO_2_ fractional laser combined with intense pulsed light (SUCRA value: 65%–70%) ranked second and third, respectively. In contrast, non-laser treatment and microneedling received the lowest rankings (SUCRA value < 20%), indicating significantly weaker efficacy in improving VSS scores compared to other interventions.

### Heterogeneity and Publication Bias

The network heterogeneity test showed low heterogeneity (I^2^ = 0%) for most direct comparisons, suggesting good data consistency. Egger regression test results (*P* = 0.6312) did not reveal significant publication bias (Supplemental Figure S1), supporting the robustness of the findings.

## Discussion

This study evaluated the efficacy of combined CO_2_ fractional laser with pulsed dye laser compared to single laser therapy and no laser treatment on hypertrophic scarring by reticulated meta-analysis system. The results showed that the combined treatment regimen was significantly superior to other interventions in terms of SMD and treatment ranking, providing an important evidence-based basis for clinical practice.

Combining the FCO_2_L with the PDL is significantly more effective than a single therapy, a result that may result from the synergistic mechanism of action of the two lasers: the FCO_2_L promotes collagen remodeling and epidermal regeneration by inducing microthermal injury, while the PDL targets the vasculature in the scar by selective photothermal action, reducing the release of inflammatory mediators and vasculoproliferation. The combination of the two therapies may simultaneously target multiple pathological aspects of scar formation (e.g., collagen metabolic imbalance, abnormal vascular proliferation, and chronic inflammation), resulting in a superimposed or complementary effect [21]. This finding is consistent with the “multi-targeted intervention” strategy proposed in previous studies and suggests the potential benefits of combination therapy in diseases with complex pathological mechanisms [4, 27].

Although both FCO_2_L monotherapy (SMD:  − 1.99) and PDL monotherapy (SMD:  − 1.42) showed superior efficacy to no-laser therapy, their effect sizes were significantly lower than the combination regimen. This may reflect the fact that monotherapy only partially intervenes in the pathological process of scar formation. The FCO_2_L improves collagen structure but has limited control of vascular abnormalities or inflammation, whereas the pulsed dye laser inhibits vascular proliferation but promotes epidermal remodeling to a lesser extent [28, 29]. This result suggests that FCO_2_L should be preferred in clinical practice when a single therapy is required due to equipment or patient tolerance, but their efficacy may not fully meet the treatment needs of patients with severe scarring.

SUCRA probability analysis showed that the combined FCO_2_L with PDL had the highest combined ranking (SUCRA >80%), further supporting its use as a first-line intervention option. However, it is important to note that the ranking results are dependent on direct and indirect comparative evidence from existing studies, and the stability of the efficacy of some of the combined regimens (e.g., FCO_2_L + IPL) needs to be validated in larger samples due to the wide confidence intervals (SMD:  − 3.14, 95% CI:  − 5.52,  − 0.77). In addition, microneedling and no-laser therapy had the lowest rankings, consistent with the limited role of microneedling in keloid treatment reported in previous studies, suggesting that it may be more suitable for mild keloid or adjunctive treatment [30–32].

This network meta-analysis focuses on hypertrophic scars. Although both hypertrophic scars and keloids clinically manifest as excessive scar tissue proliferation, they exhibit key differences in pathophysiology, behavior, and treatment response. Keloids are characterized by invasive growth beyond the original wound margins and a high recurrence rate, with typically more persistent fibroblast proliferation and collagen synthesis dysregulation [33, 34]. Although this study demonstrates that combined laser therapy (FCO_2_L + PDL) shows advantages in treating hypertrophic scars by targeting collagen remodeling and vascular abnormalities, these findings cannot be directly extrapolated to keloids. The more active biological behavior of keloids may elicit different responses to single or combined laser treatments. Scattered studies suggest that laser therapy (particularly FCO_2_L) combined with intralesional corticosteroid injections may offer some efficacy for keloids, but conclusions are inconsistent [35, 36]. Therefore, we recommend strictly limiting the interpretation of this study’s conclusions to the hypertrophic scar population. There is an urgent need for well-designed randomized controlled trials specifically evaluating the efficacy and safety of combined laser therapies in keloids to determine their practical value in this more challenging condition.

This study has the following limitations: firstly, the number of studies included and sample size. Despite our comprehensive search, high-quality RCTs meeting strict PICO criteria for head-to-head comparisons of combined versus single laser therapies remain relatively limited. This results in relatively weak direct evidence for certain treatment comparisons (particularly some combination therapies), with greater uncertainty in their efficacy estimates (reflected in wider confidence intervals). Secondly, the included studies exhibited differences in laser treatment parameters (e.g., energy, density, pulse width), treatment intervals, and total course duration, as well as the etiology and duration of scars. Although statistical heterogeneity tests (I^2^) indicated low overall heterogeneity, these clinical variations may limit the generalizability of the results. Thirdly, the studies included in this analysis did not stratify or adjust the efficacy data according to skin types (such as Fitzpatrick classification). It is known that skin type may affect the energy absorption of lasers, thermal effects, and the risk of adverse reactions such as pigmentation abnormalities, thereby potentially regulating the therapeutic effect. The patients included in our studies mainly came from Asia, the Middle East, and Europe and America, which indicates the diversity of skin types. However, the lack of relevant data limits our understanding of the impact of this important factor. Future research should systematically record and evaluate the impact of skin types on the efficacy and safety of laser combination treatment regimens to provide more individualized and meaningful evidence. Fourth, this study primarily relied on observer-rated VSS scores. Although VSS is widely used, its subjective nature may introduce measurement bias. Additionally, the lack of long-term follow-up data (e.g., recurrence rates >1 year) and patient-reported outcomes (e.g., scar-related quality of life) limits the assessment of treatment’s long-term efficacy. Furthermore, within the evidence network of this study, certain interventions (e.g., FCO_2_L + IPL) were linked to placebo through only a single study, limiting the robustness of their effect estimates.

To further clarify the advantages of combination laser therapy, it is recommended that more high-quality randomized controlled trials comparing combination therapy with monotherapy in a head-to-head manner be conducted, and that treatment parameters be harmonized to enhance comparability of results. In addition, it is important to explore the differences in efficacy of different laser combinations (e.g., FCO_2_L in combination with other wavelengths), to optimize the treatment intervals and duration, and to assess the long-term efficacy (e.g., recurrence rate beyond 1 year). Mechanistic studies can deeply analyze the molecular mechanism of combined therapies, such as the balance of collagen synthesis and degradation, and the regulation of inflammatory factors, in order to provide a more solid theoretical basis.

## Conclusion

Combined FCO_2_L and PDL showed significant advantages in improving hypertrophic scarring, with effect sizes and probability rankings supporting their use as the optimal treatment option. Despite the effectiveness of single laser treatment, the combined strategy achieved a more comprehensive pathological intervention through multimedia synergy. Future high-quality studies and long-term follow-up are needed to further validate its efficacy and optimize treatment parameters to enhance clinical applicability.

## Supplementary Information

Below is the link to the electronic supplementary material.

Supplementary file1 (TIF 77 KB)

Supplementary file2 (TIF 296 KB)

Supplementary file3 (TIF 68 KB)

Supplementary file4 (DOCX 19 KB)

Supplementary file5 (DOCX 12 KB)

## Data Availability

All data generated or analyzed in this study are included in the present manuscript.

## References

[1] Finnerty CC, Jeschke MG, Branski LK, Barret JP, Dziewulski P, Herndon DN. Hypertrophic scarring: the greatest unmet challenge after burn injury. Lancet. 2016;388(10052):1427–36. 27707499 10.1016/S0140-6736(16)31406-4 PMC5380137

[2] Tuan TL, Nichter LS. The molecular basis of keloid and hypertrophic scar formation. Mol Med Today. 1998;4(1):19–24. 9494966 10.1016/S1357-4310(97)80541-2

[3] Zhang Y, Ye R, Dong J, Bai Y, He Y, Ni W, et al. Efficacy and safety of ablative CO(2) fractional laser and narrowband intense pulsed light for the treatment of hypertrophic scars: a prospective, randomized controlled trial. J Dermatolog Treat. 2023;34(1):2202287. 37070799 10.1080/09546634.2023.2202287

[4] Kwon HH, Yang SH, Lee J, Park BC, Park KY, Jung JY, et al. Combination treatment with human adipose tissue stem cell-derived exosomes and fractional CO2 laser for acne scars: a 12-week prospective, double-blind, randomized, split-face study. Acta Derm Venereol. 2020;100(18):adv00310. 33073298 10.2340/00015555-3666 PMC9309822

[5] Leszczynski R, da Silva CA, Pinto A, Kuczynski U, da Silva EM. Laser therapy for treating hypertrophic and keloid scars. Cochrane Database Syst Rev. 2022;9(9):Cd011642. 36161591 10.1002/14651858.CD011642.pub2 PMC9511989

[6] Kang BY, Ibrahim SA, Weil A, Reynolds KA, Johnson T, Wilson S, et al. Treatment of surgical scars with combination pulsed dye and fractional nonablative laser: a randomized controlled trial. Ann Surg. 2022;276(6):975–80. 35081564 10.1097/SLA.0000000000005377

[7] Cohen JL, Geronemus R. Safety and efficacy evaluation of pulsed dye laser treatment, CO(2) ablative fractional resurfacing, and combined treatment for surgical scar clearance. J Drugs Dermatol. 2016;15(11):1315–9. 28095541

[8] Chi H, Peng H, Zhao X, Zhou G, Shen L, Cai M. The effectiveness of 595-nm pulsed dye laser for the treatment of bilateral cleft-lip scars in Asian patients: a 6-month prospective, randomized self-controlled trial. Adv Wound Care (New Rochelle). 2024;13(6):322–8. 38258794 10.1089/wound.2023.0106

[9] Zhou C, Yao M, Chen W, Zhang L, Zhang L, Chen M, et al. Treatment of erythematous acne scars using 595-nm pulsed dye laser combined with 1565-nm ResurFX nonablative fractional laser. J Cosmet Dermatol. 2024;23(6):2015–21. 38426374 10.1111/jocd.16235

[10] Kivi MK, Jafarzadeh A, Hosseini-Baharanchi FS, Salehi S, Goodarzi A. The efficacy, satisfaction, and safety of carbon dioxide (CO2) fractional laser in combination with pulsed dye laser (PDL) versus each one alone in the treatment of hypertrophic burn scars: a single-blinded randomized controlled trial. Lasers Med Sci. 2024;39(1):69. 38376542 10.1007/s10103-024-03976-6

[11] Tawaranurak N, Pliensiri P, Tawaranurak K. Combination of fractional carbon dioxide laser and topical triamcinolone vs intralesional triamcinolone for keloid treatment: A randomised clinical trial. Int Wound J. 2022;19(7):1729–35. 35166029 10.1111/iwj.13775 PMC9615274

[12] Loef M, Walach H, Schmidt S. Interrater reliability of ROB2 - an alternative measure and way of categorization. J Clin Epidemiol. 2022;142:326–7. 34509629 10.1016/j.jclinepi.2021.09.003

[13] Minozzi S, Gonzalez-Lorenzo M, Cinquini M, Berardinelli D, Cagnazzo C, Ciardullo S, et al. Adherence of systematic reviews to Cochrane RoB2 guidance was frequently poor: a meta epidemiological study. J Clin Epidemiol. 2022;152:47–55. 36156301 10.1016/j.jclinepi.2022.09.003

[14] Cooper LE, Nuutila K, Kemp Bohan PM, Diaz V, Batchinsky M, Carlsson AH, et al. Analysis of the utility of CO2 and pulse-dye lasers together and separately in the treatment of hypertrophic burn scars. Ann Plast Surg. 2022;89(2):166–72. 35943226 10.1097/SAP.0000000000003240

[15] Esmat S, Shokeir HA, Samy NA, Mahmoud SB, Sayed S, Shaker E, et al. Automated microneedling versus fractional CO2 laser in treatment of traumatic scars: a clinical and histochemical study. Dermatol Surg. 2021;47(11):1480–5. 34468410 10.1097/DSS.0000000000003227

[16] Lewis CJ, Douglas H, Martin L, Deng Z, Melton P, Fear MW, et al. Carbon dioxide laser treatment of burn-related scarring: Results of the ELIPSE (Early Laser Intervention Promotes Scar Evolution) prospective randomized controlled trial. J Plast Reconstr Aesthet Surg. 2023;84:368–76. 37393760 10.1016/j.bjps.2023.06.012

[17] Keaney TC, Tanzi E, Alster T. Comparison of 532 nm potassium titanyl phosphate laser and 595 nm pulsed dye laser in the treatment of erythematous surgical scars: a randomized, controlled, open-label study. Dermatol Surg. 2016;42(1):70–6. 26673432 10.1097/DSS.0000000000000582

[18] Ouyang HW, Li GF, Lei Y, Gold MH, Tan J. Comparison of the effectiveness of pulsed dye laser vs pulsed dye laser combined with ultrapulse fractional CO(2) laser in the treatment of immature red hypertrophic scars. J Cosmet Dermatol. 2018;17(1):54–60. 29392869 10.1111/jocd.12487

[19] Daoud AA, Gianatasio C, Rudnick A, Michael M, Waibel J. Efficacy of combined Intense Pulsed Light (IPL) with fractional CO(2)-laser ablation in the treatment of large hypertrophic scars: a prospective, randomized control trial. Lasers Surg Med. 2019;51(8):678–85. 31090087 10.1002/lsm.23092

[20] Tawfic SO, El-Tawdy A, Shalaby S, Foad A, Shaker O, Sayed SS, et al. Evaluation of fractional CO(2) versus long pulsed Nd:YAG lasers in treatment of hypertrophic scars and keloids: a randomized clinical trial. Lasers Surg Med. 2020;52(10):959–65. 32293045 10.1002/lsm.23249

[21] Keshk ZS, Salah MM, Samy NA. Fractional carbon dioxide laser treatment of hypertrophic scar clinical and histopathological evaluation. Lasers Med Sci. 2025;40(1):137. 40069407 10.1007/s10103-025-04371-5 PMC11897074

[22] Tawfic SO, Hassan AS, El-Zahraa Sh Aly F, Elbendary A, Shaker OG, AlOrbani AM. Fractional microneedle radiofrequency versus fractional carbon dioxide laser in the treatment of postburn hypertrophic scars. Lasers Surg Med. 2022;54(8):1089–98. 35900305 10.1002/lsm.23589

[23] Allison KP, Kiernan MN, Waters RA, Clement RM. Pulsed dye laser treatment of burn scars. alleviation or irritation? Burns. 2003;29(3):207–13. 12706612 10.1016/s0305-4179(02)00280-2

[24] Yang Q, Ma Y, Zhu R, Huang G, Guan M, Avram MM, et al. The effect of flashlamp pulsed dye laser on the expression of connective tissue growth factor in keloids. Lasers Surg Med. 2012;44(5):377–83. 22539077 10.1002/lsm.22031

[25] Pongcharoen P, Pongcharoen B, Disphanurat W. The effectiveness of a 595 nm pulsed-dye-laser in the treatment of surgical scars following a knee arthroplasty. J Cosmet Laser Ther. 2019;21(6):352–6. 31462121 10.1080/14764172.2019.1661488

[26] Azzam OA, Bassiouny DA, El-Hawary MS, El Maadawi ZM, Sobhi RM, El-Mesidy MS. Treatment of hypertrophic scars and keloids by fractional carbon dioxide laser: a clinical, histological, and immunohistochemical study. Lasers Med Sci. 2016;31(1):9–18. 26498451 10.1007/s10103-015-1824-4

[27] Karampinis E, Georgopoulou KE, Goudouras G, Lianou V, Kampra E, Roussaki Schulze AV, et al. Laser-induced Koebner-related skin reactions: a clinical overview. Medicina (Kaunas). 2024. 10.3390/medicina60071177. 39064606 10.3390/medicina60071177 PMC11278978

[28] Prignano F, Bonciani D, Campolmi P, Cannarozzo G, Bonan P, Lotti T. A study of fractional CO₂ laser resurfacing: the best fluences through a clinical, histological, and ultrastructural evaluation. J Cosmet Dermatol. 2011;10(3):210–6. 21896133 10.1111/j.1473-2165.2011.00571.x

[29] Glassberg E, Lask GP, Tan EM, Uitto J. Cellular effects of the pulsed tunable dye laser at 577 nanometers on human endothelial cells, fibroblasts, and erythrocytes: an in vitro study. Lasers Surg Med. 1988;8(6):567–72. 3210881 10.1002/lsm.1900080605

[30] Sitohang IBS, Sirait SAP, Suryanegara J. Microneedling in the treatment of atrophic scars: a systematic review of randomised controlled trials. Int Wound J. 2021;18(5):577–85. 33538106 10.1111/iwj.13559 PMC8450803

[31] Claytor RB, Sheck CG, Chopra V. Microneedling outcomes in early postsurgical scars. Plast Reconstr Surg. 2022;150(3):557e-e561. 35759632 10.1097/PRS.0000000000009466

[32] Li H, Jia B, Zhang X. Comparing the efficacy and safety of microneedling and its combination with other treatments in patients with acne scars: a network meta-analysis of randomized controlled trials. Arch Dermatol Res. 2024;316(8):505. 39110247 10.1007/s00403-024-03256-x

[33] Menashe S, Heller L. Keloid and hypertrophic scars treatment. Aesthetic Plast Surg. 2024;48(13):2553–60. 38453710 10.1007/s00266-024-03869-7

[34] Hirsch Y, Waterman CL, Haber R. Pediatric keloids and review of the efficacy of current treatment modalities. Dermatol Surg. 2023;49(7):669–74. 37134240 10.1097/DSS.0000000000003815

[35] Goel J, Singh VK, Haq A, Pp S, Verma S. Split-scar technique to assess the efficacy of Er:YAG laser with intralesional triamcinolone combination on post-burn scars-a double blind, parallel, two-arm randomized controlled trial. Burns. 2025;51(8):107644. 40834485 10.1016/j.burns.2025.107644

[36] Krishna PS, Gopal KVT, Ananditha K, Reddy VST, Rao TN. A randomized study to evaluate the efficacy and adverse effects of cryotherapy combined with intralesional steroids, intralesional bleomycin combined with steroids and fractional CO(2) laser in keloids. Indian Dermatol Online J. 2025;16(3):407–13. 40395571 10.4103/idoj.idoj_789_24 PMC12088498

