# Supplementary figures and images for: Comparative Efficacy of Combined Carbon Dioxide Fractional Laser and Pulse Dye Laser versus Monotherapy for Hypertrophic Scars: A Network Meta-Analysis of Randomized Controlled Trials
Source: Aesthetic Plast Surg. 2026 Apr 15;50(11):4450–60. doi: 10.1007/s00266-026-05829-9 (PMC13314835; doi:10.1007/s00266-026-05829-9)

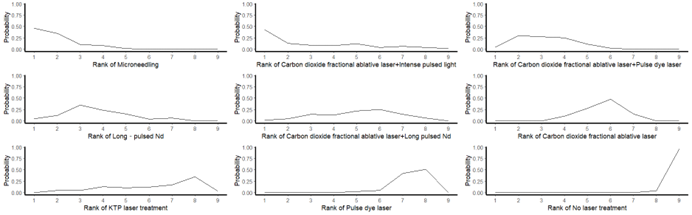

Supplement: Supplementary file 1 — Supplementary file1 (TIF 77 KB) [file 266_2026_5829_MOESM1_ESM.tif]

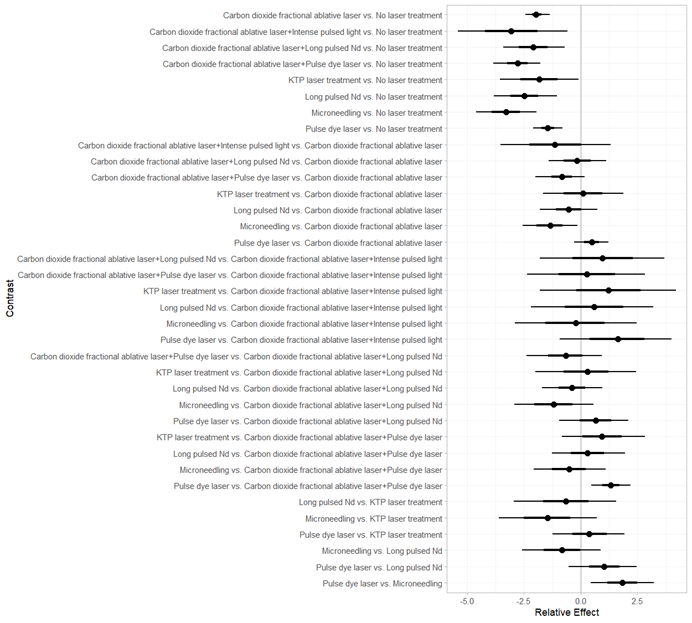

Supplement: Supplementary file 2 — Supplementary file2 (TIF 296 KB) [file 266_2026_5829_MOESM2_ESM.tif]

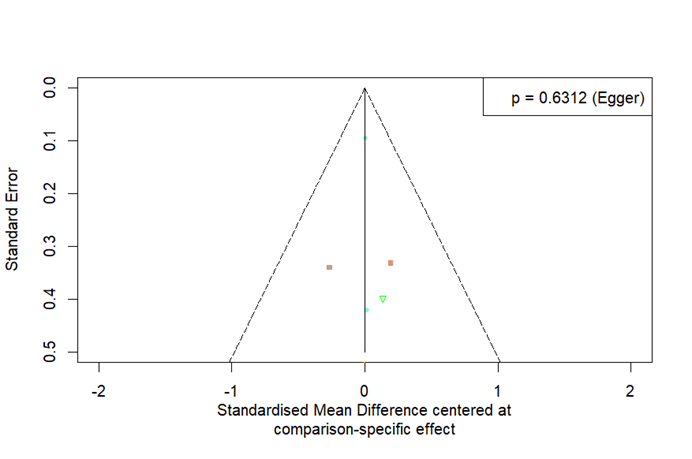

Supplement: Supplementary file 3 — Supplementary file3 (TIF 68 KB) [file 266_2026_5829_MOESM3_ESM.tif]
