# Supplementary material for: Comparative Efficacy of Combined Carbon Dioxide Fractional Laser and Pulse Dye Laser versus Monotherapy for Hypertrophic Scars: A Network Meta-Analysis of Randomized Controlled Trials
Source: Aesthetic Plast Surg. 2026 Apr 15;50(11):4450–60. doi: 10.1007/s00266-026-05829-9 (PMC13314835; doi:10.1007/s00266-026-05829-9)
Supplement: Supplementary file 4 — Supplementary file4 (DOCX 19 KB) [file 266_2026_5829_MOESM4_ESM.docx]

**Supplement table**

**Table S1 Search strategy**

**PubMed**

| **Set** | **Search terms** | **Search type** | **Results** |
| --- | --- | --- | --- |
| #1 | "Cicatrix, Hypertrophic"[Mesh] OR "Cicatrices, Hypertrophic"[tiab] OR "Hypertrophic Cicatrix"[tiab] OR "Scars, Hypertrophic"[tiab] OR "Hypertrophic Scar*"[tiab] OR "HS"[tiab] OR "Keloid*"[Mesh] | Advanced | 61,738 |
| #2 | "Fractional Carbon Dioxide Laser "[tiab] OR "fractional CO2 laser"[tiab] OR "FCO^2^L"[tiab] | Advanced | 746 |
| #3 | "Lasers, Dye/therapeutic use"[Mesh] OR "Dye Laser*"[tiab] OR "Pulsed Dye Lasers"[tiab] OR "Pulsed Dye Laser"[tiab] OR "PDL"[tiab] | Advanced | 9,353 |
| #4 | "Randomized controlled trial[Publication Type]" OR "RCT[Publication Type]" | Advanced | 638,452 |
| #5 | #1 AND (#2 OR #3) AND #4 | Advanced | 35 |

**Embase**

| **Set** | **Search terms** | **Search type** | **Results** |
| --- | --- | --- | --- |
| #1 | 'hypertrophic scar'/exp OR 'cicatrix, hypertrophic':ti,ab,kw OR 'hyper-trophic scar':ti,ab,kw OR 'hyper-trophic scarring':ti,ab,kw OR 'hypertrophic cicatrices':ti,ab,kw OR 'hypertrophic cicatrisation':ti,ab,kw OR 'hypertrophic cicatrix':ti,ab,kw OR 'hypertrophic cicatrization':ti,ab,kw OR 'hypertrophic scarring':ti,ab,kw OR 'hypertrophic scar':ti,ab,kw | Advanced | 8,640 |
| #2 | 'fractional carbon dioxide laser therapy'/exp OR 'fractional co2 laser':ti,ab,kw OR "fco2l":ti,ab,kw | Advanced | 483 |
| #3 | 'pulsed dye laser'/exp OR 'dye laser*':ti,ab,kw | Advanced | 5,689 |
| #4 | 'randomized controlled trial'/exp OR 'randomised controlled study ':ti,ab,kw | Advanced | 930,615 |
| #5 | #1 AND (#2 OR #3) AND #4 | Advanced | 37 |

**Cochrane library**

| **Set** | **Search terms** | **Search type** | **Results** |
| --- | --- | --- | --- |
| #1 | MeSH descriptor: [Cicatrix, Hypertrophic] explode all trees | Advanced | 326 |
| #2 | (hypertrophic scar):ti,ab,kw OR (cicatrix, hypertrophic):ti,ab,kw OR (hypertrophic cicatrization):ti,ab,kw OR (hyper-trophic scarring):ti,ab,kw OR (hypertrophic cicatrices):ti,ab,kw | Advanced | 866 |
| #3 | #1 OR #2 | Advanced | 866 |
| #4 | (Fractional Carbon Dioxide Laser):ti,ab,kw OR (fractional co2 laser):ti,ab,kw OR (FCO2L):ti,ab,kw | Advanced | 756 |
| #5 | MeSH descriptor: [Lasers, Dye] explode all trees | Advanced | 130 |
| #6 | (Tunable Dye Lasers):ti,ab,kw OR (pulsed dye laser):ti,ab,kw | Advanced | 442 |
| #7 | #5 OR #6 | Advanced | 447 |
| #8 | (randomised controlled study):ti,ab,kw OR ("randomized-controlled trial"):ti,ab,kw OR (RCT):ti,ab,kw | Advanced | 852,353 |
| #9 | #3 AND (#4 OR #7) AND #8 | Advanced | 74 |

**Web of Science**

| **Set** | **Search terms** | **Search type** | **Results** |
| --- | --- | --- | --- |
| #1 | TS=('hypertrophic scar' OR 'cicatrix, hypertrophic' OR 'hyper-trophic scar' OR 'hyper-trophic scarring' OR 'hypertrophic cicatrices' OR 'hypertrophic cicatrisation' OR 'hypertrophic cicatrix'OR 'hypertrophic cicatrization' OR 'hypertrophic scarring' OR 'hypertrophic scar') | Advanced | 10,262 |
| #2 | TS=('fractional carbon dioxide laser therapy' OR 'fractional co2 laser' "fco2l") | Advanced | 1,027 |
| #3 | TS=("Lasers, Dye/therapeutic use"OR "Dye Laser*" OR "Pulsed Dye Lasers" OR "Pulsed Dye Laser" OR "PDL") | Advanced | 20,022 |
| #4 | TS=("randomised controlled study" OR "randomized-controlled trial" OR "RCT") | Advanced | 291,145 |
| #5 | #1 AND (#2 OR #3) AND #4 | Advanced | 47 |
