# Supplementary material for: Comparative Efficacy of Combined Carbon Dioxide Fractional Laser and Pulse Dye Laser versus Monotherapy for Hypertrophic Scars: A Network Meta-Analysis of Randomized Controlled Trials
Source: Aesthetic Plast Surg. 2026 Apr 15;50(11):4450–60. doi: 10.1007/s00266-026-05829-9 (PMC13314835; doi:10.1007/s00266-026-05829-9)
Supplement: Supplementary file 5 — Supplementary file5 (DOCX 12 KB) [file 266_2026_5829_MOESM5_ESM.docx]

**Supplement Figure legends**

**Figure S1 SUCRA Sorting Chart.**

**Figure S2 Node Segmentation Chart.**

**Figure S3 Funnel Chart.**
